# Nutritional risk and HbA1c as critical risk factors and predictors of opportunistic infections in HIV-DM comorbid patients: a retrospective cross-sectional study

**DOI:** 10.3389/fendo.2024.1527936

**Published:** 2025-01-10

**Authors:** Qiong’e Zhu, Fengjiao Gao, Xiaoxia Ren, Rui Li, Jun Kang, Maoquan Li, Dafeng Liu

**Affiliations:** ^1^ The First Ward of Internal Medicine, Public Health Clinical Center of Chengdu, Chengdu, China; ^2^ Department of Public Health, Chengdu Medical College, Chengdu, China; ^3^ Office of the Party Committee (Administration), Neijiang Health Vocational College, Neijiang, China

**Keywords:** HIV, diabetes, nutritional risk, glycosylated hemoglobin, opportunistic Infections

## Abstract

**Background:**

The clinical characteristics and risk factors for opportunistic infections in HIV patients with concomitant diabetes mellitus are unclear and worth studying. Explore the risk factors and construct a predictive model for opportunistic infections in HIV-DM patients.

**Methods:**

Clinical data were retrospectively collected from 1,669 HIV-DM admitted to the Public Health Clinical Center of Chengdu from December 2018 to November 2023.Based on the occurrence of opportunistic infections, the patients were divided into two groups. Comparative analyses were conducted to assess nutritional status, plasma glucose levels, comorbidities of chronic noncommunicable diseases, and immune status. We applied univariate logistic regression and LASSO regression to screen independent variables. Multivariable logistic regression analysis was performed to identify risk factors and establish a prediction model for opportunistic infections.

**Results:**

In our study population of 1,669 patients (median age was 59 years, 83.22% male), 868 developed opportunistic infections, while the remaining 801 patients did not develop opportunistic infections. Additionally, 1,598 patients survived, and 71 patients died. The opportunistic infection group had a greater median age, lower median BMI, longer median length of hospital stay and greater number of deaths. Logistic regression analysis identified nutritional risk, duration of HIV infection, HbA1c, albumin, and CD4+ T cell counts as significant risk factors and predictors of opportunistic infections. Nutritional risk (OR=2.888) and HbA1c showed positive associations (*P*<0.001), while duration of HIV infection, albumin, and CD4+ T cell counts demonstrated negative associations with infection risk (*P*<0.05). The comprehensive model evaluation, based on receiver operating characteristic (ROC) curve, calibration curve, and decision curve analysis (DCA), validated the acceptable predictive performance of our model.

**Conclusion:**

This study identified nutritional risk, duration of HIV infection, HbA1c and albumin as significant risk factors and predictors of opportunistic infections in HIV-DM patients, further highlighting the importance of nutritional screening and good glucose control.

## Introduction

In recent years, the application of highly active antiretroviral therapy (HAART) has significantly improved the life expectancy of patients who are human immunodeficiency virus (HIV)-positive, bringing their lifespan closer to that of the general population (1). However, this advancement has concurrently led to a rising prevalence of noncommunicable diseases, including chronic metabolic and cardiovascular diseases such as diabetes mellitus. Diabetes mellitus is relatively common among individuals living with human immunodeficiency virus (2). The use of highly active antiretroviral therapy appears to exacerbate metabolic and endocrine dysfunctions, including insulin resistance, dyslipidemia, and lipodystrophy, thus contributing to an increased disease burden (3–5). More significantly, the coexistence of human immunodeficiency virus infection and diabetes mellitus can intensify immune dysfunction and inflammatory responses (6), consequently increasing susceptibility to opportunistic infections.

Opportunistic infections refer to a spectrum of infections caused by organisms (bacterial, viral, fungal, and protozoal pathogens) that are generally harmless in individuals with normal immune function but manifest as significant infections in immunocompromised hosts, most notably in HIV-infected individuals with reduced CD4+ T-lymphocyte counts (7). Despite opportunistic infections remaining the leading cause of mortality and substantial economic burden in HIV patients, the implementation of preventive strategies has successfully improved survival outcomes while reducing healthcare expenditures (8).The risk of opportunistic infections can be mitigated not only through conventional preventive measures such as co-infection screening, antimicrobial prophylaxis, vaccinations, and exposure risk reduction (9), but also through comprehensive clinical management incorporating nutritional risk assessment, immunological monitoring, and systematic disease evaluation.

Individuals with human immunodeficiency virus infection who develop opportunistic infections frequently demonstrate compromised nutritional and immunological status, characterized by lower BMI,CD4+ T-cell counts and reduced baseline hemoglobin levels (10–13).Poor glycemic homeostasis can synergistically amplify immune system dysfunction and inflammatory cascades, consequently heightening the risk of opportunistic infections (6). While chronic comorbidities have been established as risk factors for opportunistic infections (14), the relative contribution of particular comorbid conditions to infection susceptibility remains to be determined.

Although extensive research has been conducted on opportunistic infections in patients with human immunodeficiency virus, these studies have paid limited attention to the concurrent presence of diabetes mellitus. In the context of this dual diagnosis, elucidating the complex interplay between nutritional status, glycemic level, and immune function, additional comorbid conditions, and the development of opportunistic infections remains a significant clinical and research challenge. The objective of this study was to investigate the impact of nutritional status, glycemic level, immune function and multiple comorbidities on the incidence of opportunistic infections in human immunodeficiency virus infection and diabetes mellitus (HIV-DM) patients.

## Methods

### Study population and eligibility criteria

In this retrospective study, we analyzed data from 1,669 patients who were hospitalized at the Public Health Clinical Center of Chengdu between December 2018 and November 2023.The inclusion criteria were as follows: 1. Age ≥ 18 years; 2. Met the diagnostic criteria for HIV and diabetes mellitus. Exclusion criteria: 1.Hospitalization duration ≤ 1 day;2.All baseline data and laboratory diagnostic indicators are missing.

### Disease diagnosis criteria

The diagnosis of AIDS and AIDS-related opportunistic infections conformed to the criteria set forth in the “Chinese Guidelines for the Diagnosis and Treatment of HIV/AIDS (2018 Edition) (15). “ The diagnosis and classification criteria for diabetes adhered to those outlined in the “Chinese Guidelines for the Prevention and Treatment of Type 2 Diabetes in China (2020 Edition) (16). “

### Grouping standards

The 1,669 HIV-infected patients with diabetes were categorized into two sets of groups based on different criteria. Patients were classified into the opportunistic infections group (OIs group, n=868) and the non-opportunistic infections group (non-OIs group, n=801) according to the presence or absence of opportunistic infections. (**Tables 1**, **2**).

**Table 1:** Comparison of baseline conditions between the OIs group and the non-OIs group.

Variables	Opportunistic Infectious Diseases		$\chi^{2}$	*P*
	OIs (n=868)	Non-OIs (n=801)		
Age(year), [M(IQR)]	61(53,69)	58(50,67)		<0.001
BMI(kg/m^2^),[M(IQR)]	22.03(19.72,24.03)	22.96(20.4,25.51)		<0.001
Duration of hospitalization (day), [M (IQR)]	15(9,23)	13(7,20)		<0.001
Albumin	32.45(28.2,37.8)	37.2(31.9,41.9)		<0.001
Total protein	65.5(59.5,71.6)	68.2(62.2,73.6)		<0.001
Lymphocytes	0.85(0.52,1.36)	1.18(0.83,1.60)		<0.001
CD3+T cell counts	624(345,995)	829(560,1115)		<0.001
CD4+ T cell counts	151(65,297)	261(131,445)		<0.001
CD8+ T cell counts	402(213,620)	468(302,679)		<0.001
HbA1c	7.6(6.4,9.5)	7.2(6.3,9.1)		0.003
Gender			0.013	0.908
male, n(%)	721(83.06)	668(83.40)		
female, n(%)	147(16.94)	133(16.60)		
Nutritional risk			166.97	<0.001
Without(NRS2002<3),n(%)	227(26.15)	460(57.43)		
With(NRS2002≥3),n(%)	641(73.85)	341(42.57)		
Nutritional support			12.21	<0.001
Yes, n(%)	222(25.58)	147(18.35)		
No, n(%)	646(74.42)	654(81.65)		
The occurrence of acute complications of diabetes			13.92	<0.001
Yes, n(%)	76(8.76)	33(4.12)		
No, n(%)	792(91.24)	768(95.88)		
Whether the plasma glucose levels meets the targets at hospital discharge			4.48	0.034
Yes, n(%)	351(40.44)	366(45.69)		
No, n(%)	517(59.56)	435(54.31)		
Duration of HIV infection			56.347	<0.001
<5 year,n(%)	491(56.57)	305(38.08)		
≥5 year,n(%)	377(43.43)	496(61.92)		
Duration of antiretroviral therapy			<0.001
No treatment,n(%)	202(23.27)	121(15.11)		
<1 year,n(%)	270(31.11)	173(21.60)		
1~5 year,n(%)	217(25.00)	245(30.59)		
≥5 year,n(%)	179(20.62)	262(32.71)		
Number of comorbidities			4.372	0.112
0, n(%)	399(45.97)	407(50.81)		
1, n(%)	226(26.04)	199(24.84)		
≥2, n(%)	243(28.00)	195(24.34)		
Coronary heart disease			5.625	0.018
Yes, n(%)	72(8.29)	42(5.24)		
No, n(%)	796(91.71)	759(94.76)		
stroke			7.535	0.006
Yes, n(%)	161(18.55)	108(13.48)		
No, n(%)	707(81.45)	693(86.52)		
Hyperlipidemia			6.333	0.012
Yes, n(%)	98(11.29)	125(15.61)		
No, n(%)	770(88.71)	676(84.39)		
Prognosis			22.583	<0.001
survive, n(%)	811(93.43)	787(98.25)		
death, n(%)	57(6.57)	14(1.75)		
Number of opportunistic infectious diseases				
1, n(%)	600(35.95)			
≥2, n(%)	268(16.06)			

**Table 2:** Results of multivariate logistic regression of risk factor analysis models for opportunistic infections.

Model	Variable	B	Std_Error	Wald	95% CI	*P*
Model 1	Nutritional risk	1.025	0.113	82.167	2.788 (2.233,3.479)	<0.001
	Lymphocytes	-0.34	0.083	16.749	0.712 (0.605,0.838)	<0.001
	Albumin	-0.049	0.008	35.538	0.952 (0.937,0.968)	<0.001
	HbA1c	0.066	0.021	9.877	1.069 (1.025,1.114)	0.002
Model 2	Nutritional risk	1.005	0.113	78.438	2.731 (2.187,3.411)	<0.001
	Duration of HIV infection	-0.450	0.11	16.881	0.638 (0.514,0.790)	<0.001
	Albumin	-0.043	0.008	26.465	0.958 (0.942,0.974)	<0.001
	CD4+T cell counts	-0.001	0.000	16.936	0.999 (0.998,0.999)	<0.001
Model 3	Nutritional risk	1.000	0.114	76.581	2.717 (2.172,3.399)	<0.001
	Duration of HIV infection	-0.437	0.110	15.765	0.646 (0.521,0.801)	<0.001
	HbA1c	0.059	0.021	7.561	1.060 (1.017,1.106)	0.006
	Lymphocytes	-0.188	0.094	4.038	0.828 (0.689,0.995)	0.044
	Albumin	-0.040	0.008	22.647	0.961 (0.945,0.977)	<0.001
	CD4+T cell counts	-0.001	0.000	7.725	0.999 (0.999,1.000)	0.005

### Data collection

Researchers collected data from 1,669 patients hospitalized at the Public Health Clinical Center of Chengdu between December 2018 and November 2023. The collected data included demographic details, clinical information, and laboratory results. Upon admission, the patients underwent nutritional risk assessment using the Nutritional Risk Screening 2002 (NRS2002) tool. The NRS2002 score ranges from 0 to 7, with scores ≥3 indicating risk of malnutrition and scores <3 indicating no nutritional risk (17). Non-achievement of glycemic targets at discharge was defined as elevated fasting or random plasma glucose levels above normal ranges on either the discharge day or the day prior. A database was established with stringent control measures implemented to ensure the accuracy, completeness, and authenticity of all the data. All personal identifiers have been removed from the dataset to ensure complete anonymization and protect patient privacy in accordance with ethical guidelines and data protection regulations.

### Statistical methods

Missing covariates were handled using multiple imputation. We conducted a comprehensive assessment of missing data patterns before performing multiple imputation. The proportion of missing values varied across variables (range: 0.72%-37.21%), with HbA1c having a missing rate of 37.21%. Despite this relatively higher missing rate, HbA1c was retained in the analysis given its established clinical significance and the acceptable missing rate (18), while all other variables demonstrated acceptable missing rates below 30%. The normality of continuous variables was assessed using the Shapiro-Wilk test. All continuous variables showed non-normal distribution (*P*<0.05). Based on these results, we employed non-parametric statistical methods: medians with IQRs for descriptive statistics and Mann-Whitney U tests for group comparisons. For categorical variables, chi-square tests were used for nominal variables, while rank-sum tests were applied for ordinal variables. Variable selection was conducted through univariate logistic regression and least absolute shrinkage and selection operator (LASSO) regression. LASSO regression is a regularized regression method that combines variable selection and regularization to enhance model prediction accuracy and interpretability. By adding a penalty term (L1 regularization) to the regression, LASSO effectively shrinks some coefficients to exactly zero, thereby automatically performing feature selection and producing more parsimonious models. This characteristic makes LASSO particularly valuable in scenarios with multiple potential predictors, as it helps identify the most relevant variables while reducing the risk of overfitting. Subsequently, a multivariate logistic regression model was constructed. Model performance was evaluated using receiver operating characteristic (ROC) curves. For model development, the dataset was randomly partitioned into a training set (70% of the data) and a test set (30%). The prediction model was developed using logistic regression algorithm, based on independent variables from the model with an optimal area under the curve (AUC). Model stability and generalizability were assessed through ten-fold cross-validation prior to construction. Model performance was evaluated on both training and test datasets using receiver operating characteristic curves, calibration plots, and decision curve analysis (DCA). DCA is a method for evaluating prediction models and diagnostic tests that takes into account clinical consequences. The analysis produces a ‘net benefit’ for prediction models by summing the benefits (true positives) and subtracting the harms (false positives). The DCA plots show the net benefit against threshold probability, where the threshold probability represents the probability at which a clinician would choose to treat. The analysis compares the prediction model with two extreme scenarios: treating all patients and treating no patients. A model is considered to have clinical utility if it shows higher net benefit than both the treat-all and treat-none strategies across a reasonable range of threshold probabilities. A nomogram was constructed to provide a visual representation of the prediction model. Statistical analyses were performed using R software (version 4.4.2).

### Patient and public involvement

Neither patients nor the public were involved in developing research questions or designing this study. Although all patients received verbal and written information about the study, they were not involved in participant recruitment or study conduct. The burden of participation was assessed by the research team. Patient eligibility assessment and data collection were performed by the investigators. The Ethics Committee of the Public Health Clinical Center of Chengdu waived the requirement for informed consent due to the retrospective nature of this study. All participants were informed that any published data (without personal identifiers) would be freely accessible to the public via the internet. General results will be available to participants upon request.

## Results

### Demographic and clinical characteristics of the study population

This study enrolled 1,669 patients with HIV-DM. The study population consisted primarily of middle-aged male patients. The data indicated that 52.01% of patients had opportunistic infections, with 16.02% presenting with multiple types of opportunistic infections. Comorbidities were present in 51.71% of patients, with 50.75% of these cases involving multiple concurrent conditions (**Table 1**).

**Figure 1:**
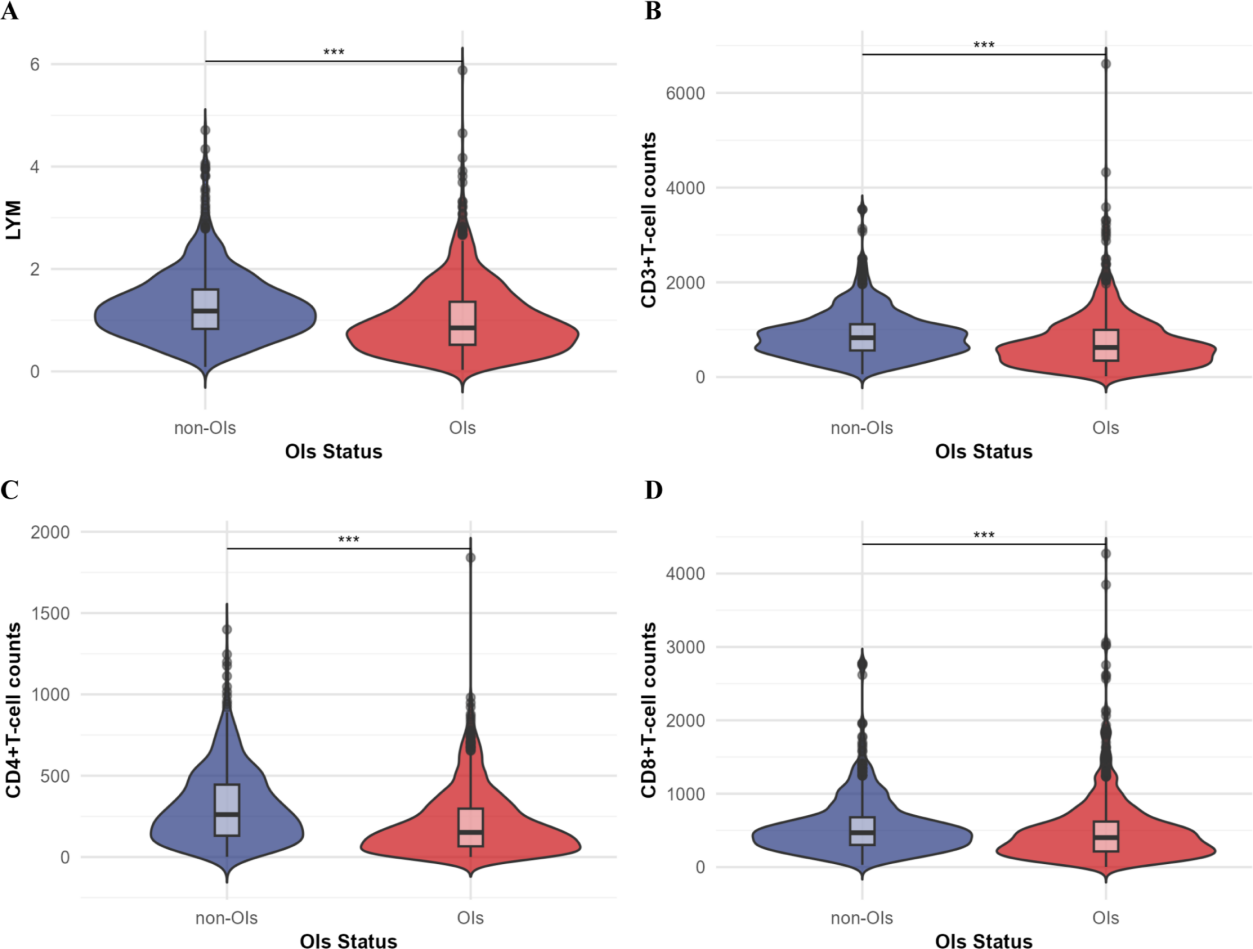
Comparison of T-cell subsets (unit: cells/ul) and lymphocyte counts (LYM) between the OIs group and the non-OIs group. **(A)** Lymphocyte counts; **(B)** CD3+ T-cell counts; **(C)** CD4+ T-cell counts; **(D)** CD8+ T-cell counts. Comparison between the two groups was performed by Mann-Whitney U test, ***P<0.001.

Statistical analyses revealed distinct clinical characteristics between opportunistic infections and non-opportunistic infections groups (**Table 1**). Patients with opportunistic infections exhibited characteristics higher age, lower body mass index, and extended hospital stays compared to those without opportunistic infections(*P*<0.001). Laboratory analyses demonstrated reduced levels of nutritional indicators in the opportunistic infections group, including albumin (ALB) and total protein, accompanied by an increased prevalence of nutritional risk. Immunological parameters differed significantly between groups. The opportunistic infections group demonstrated reduced lymphocyte counts (LYM) and lower T-cell subset levels, including CD3+T cell counts, CD4+T cell counts and CD8+T cell counts (**Figures 1A–D**).

Patients in the opportunistic infections group demonstrated impaired glycemic control, characterized by elevated glycosylated hemoglobin (HbA1c) levels and lower proportion of patients achieving glycemic targets at hospital discharge. This group also exhibited higher incidence of acute diabetic complications and increased mortality rates.

The correlations among all independent variables and key independent variables are presented in **Supplementary Figures 1**, **2**, respectively.

### Development of risk factor analysis models for opportunistic infections in HIV-DM patients

**Figure 2:**
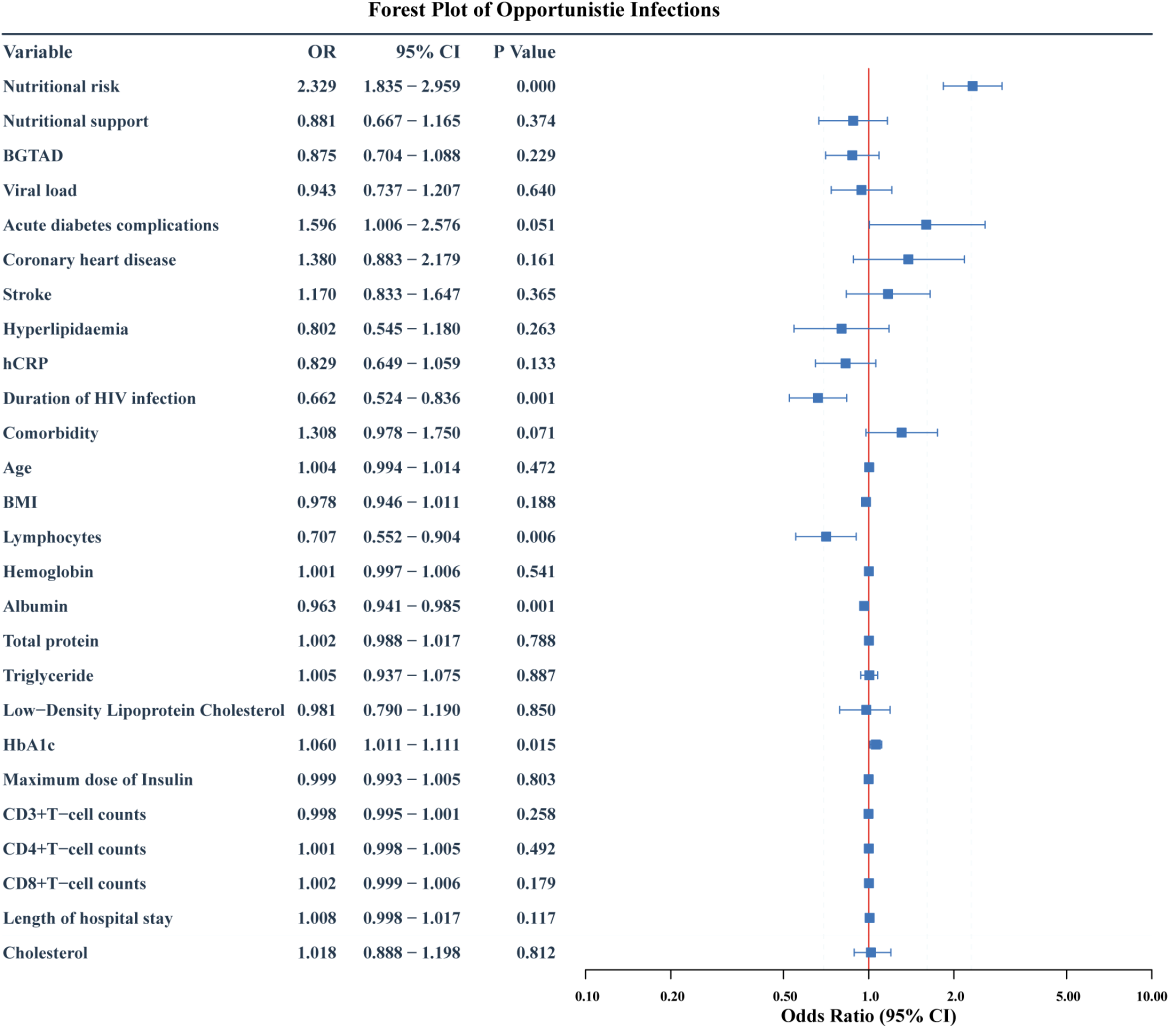
Forest plot of risk factors for opportunistic infections: initial multivariate Model 1. The forest plot shows the odds ratio (OR), 95% confidence interval (95%CI) and p value of HIV-DM patients for developing opportunistic infection after multivariate logistic regression. BGTAD, Blood glucose target achievement at discharge.

**Figure 3:**
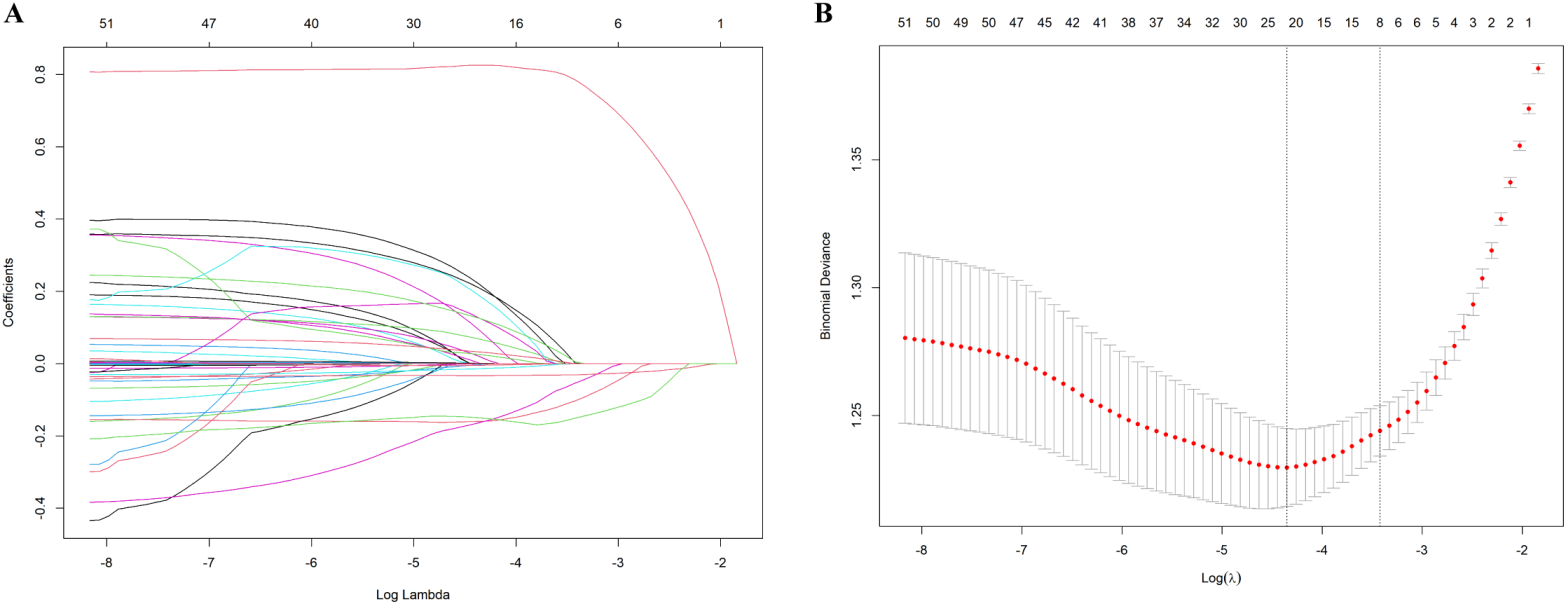
Results of the LASSO regression. **(A)** The optimal parameter λ in LASSO regression model with logλ as abscissa and regression coefficient as ordinate; **(B)** 10-fold cross-validation by minimum criteria, adjusting the parameter (λ) selection in the LASSO model, where log(λ) is the lower abscissa, binomial deviation is the ordinate, and the number of variables is the upper abscissa.

The potential risk factors for opportunistic infections were initially analyzed using univariate logistic regression and LASSO regression. The univariate analysis included 27 candidate variables (**Figure 2**). In the LASSO regression, we selected the optimal λ value within one standard deviation of the minimum binomial deviation (**Figures 3A, B**). This approach identified six significant variables including nutritional risk, duration of HIV infection, high-sensitivity C-reactive protein (h-CRP), lymphocyte counts, albumin, and CD4+ T-cell counts. Multivariate logistic regression analysis was performed on variables selected by both methods, establishing Model 1 with variables identified from univariate logistic regression and Model 2 with variables derived from LASSO regression. Variables that remained significant (*P*<0.05) in both models were then combined to create Model 3 (**Table 2**). Based on these analyses, we established three multivariate logistic regression models to identify significant risk factors. In Model 1, the presence of nutritional risk, low lymphocyte counts, low serum albumin levels and elevated HbA1c levels were identified as significant risk factors for opportunistic infections(*P*<0.05). Model 2 identified nutritional risk, duration of HIV infection, low serum albumin levels, and low CD4+ T-cell counts as significant risk factors (*P*<0.001). Model 3 integrated the consistently significant risk factors from the previous two models, further validating their associations with opportunistic infections(*P*<0.05).

### Evaluation of risk factor analysis models for opportunistic infections in HIV-DM patients

**Figure 4:**
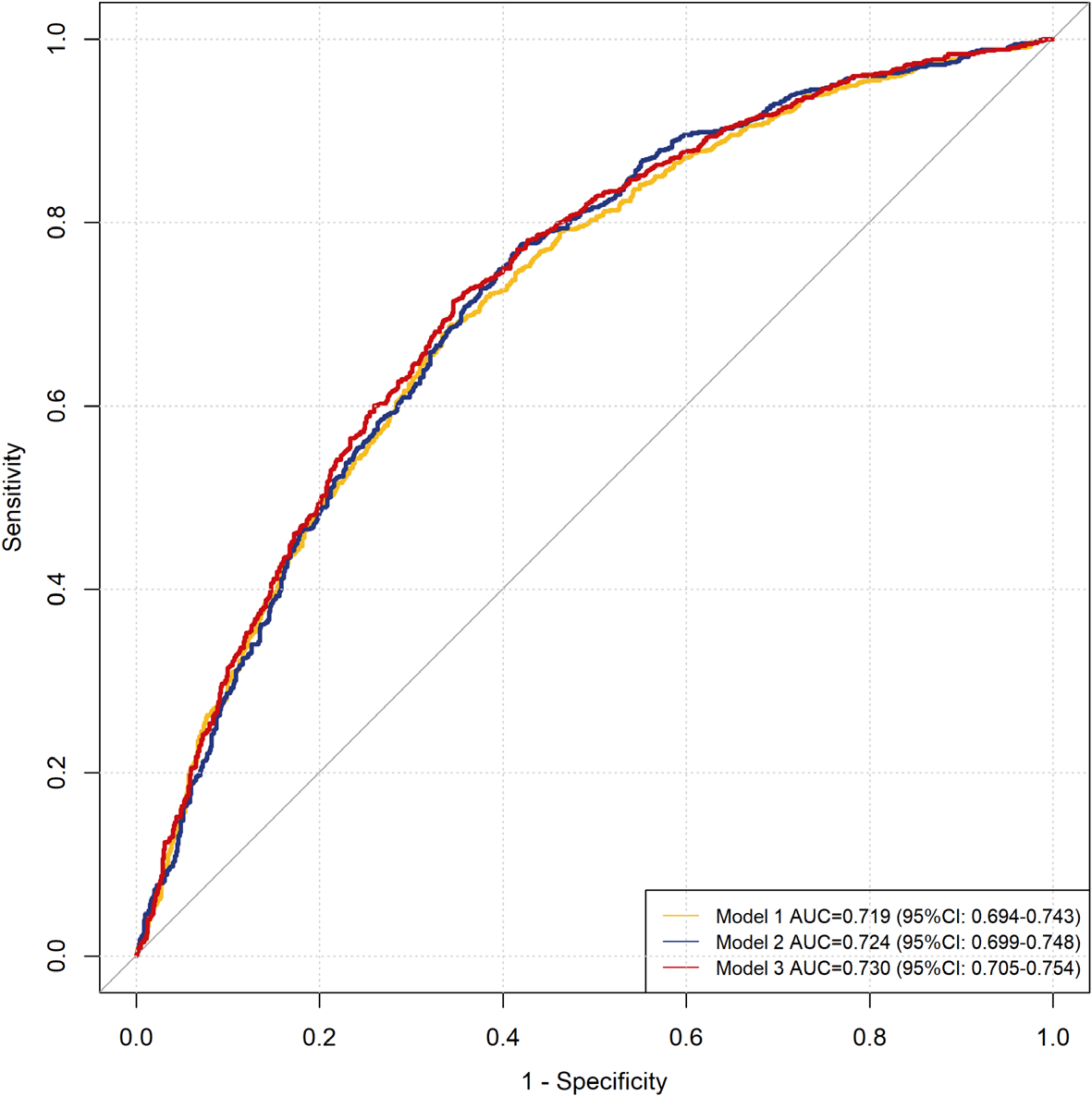
ROC curve of risk factor analysis models for opportunistic infections.

The discriminative ability of the three models was assessed using ROC curve analysis, which showed moderate performance with areas under the curve of 0.719(95%CI:0.694-0.743), 0.724(95%CI:0.699-0.748), and 0.730(95%CI:0.705-0.754) for Models 1, 2 and 3, respectively (**Figure 4**).

### Development of a predictive model for opportunistic infections in HIV-DM patients

A prediction model was developed using the six independent variables selected from Model 3, including nutritional risk, duration of HIV infection, lymphocyte counts, albumin, HbA1c and CD4+ T-cell counts. Multivariate logistic regression analysis (**Table 3**) revealed that nutritional risk (OR=2.859, 95%CI: 2.184-3.750) and HbA1c (OR=1.054, 95%CI:1.002-1.109) were positively associated with opportunistic infections (*P*<0.05). Duration of HIV infection (OR=0.576, 95%CI:0.444-0.747), albumin (OR=0.956, 95%CI:0.936, 0.976) and CD4+T cell counts (OR=0.999, 95%CI: 0.998,1.000) showed negative associations with opportunistic infections (*P*<0.05). No significant association was found between lymphocyte counts and opportunistic infections (*P*>0.05).

**Table 3:** Results of multivariate logistic regression of predictive model for opportunistic infections.

Variable	B	Std_Error	Wald	95% CI	*P*
Nutritional risk	1.050	0.138	58.064	2.859 (2.184,3.750)	<0.001
Duration of HIV infection	-0.552	0.133	17.277	0.576 (0.444,0.747)	<0.001
HbA1c	0.053	0.026	4.107	1.054 (1.002,1.109)	0.043
Lymphocytes	-0.165	0.113	2.146	0.848 (0.678,1.057)	0.143
Albumin	-0.045	0.011	18.251	0.956 (0.936,0.976)	<0.001
CD4+T cell counts	-0.001	0.000	8.379	0.999 (0.998,1.000)	0.004

### Validation of a predictive model for opportunistic infections in HIV-DM patients

In the training dataset, the model demonstrated consistency across different data partitions with an area under the curve of 0.744 ± 0.034 in ten-fold cross-validation.

**Figure 5:**
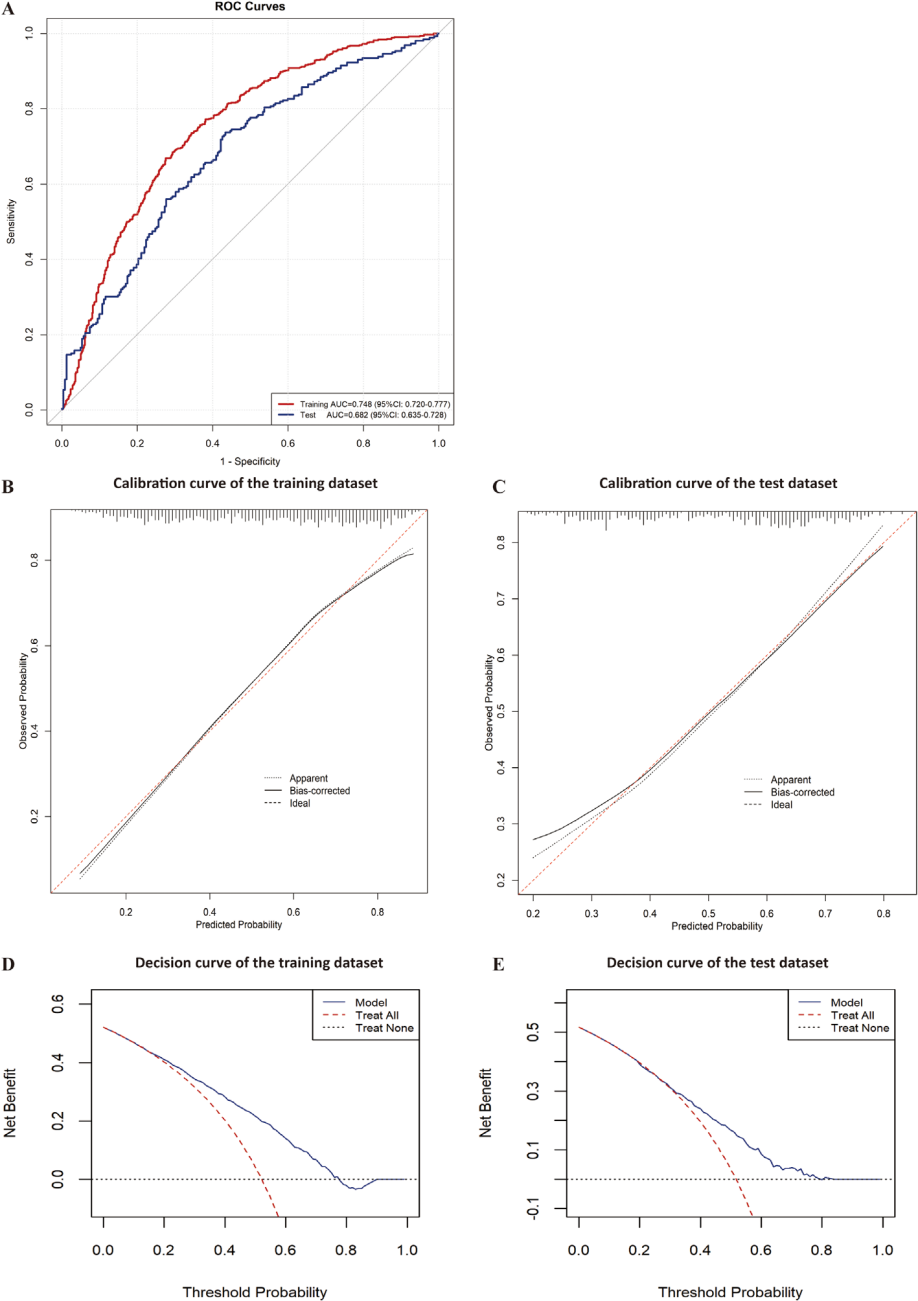
Evaluation of the predictive model for opportunistic infections. **(A)** ROC curve of the training and test datasets for the predictive model; **(B)** Calibration curve of the training dataset for the predictive model; **(C)** Calibration curve of the test dataset for the predictive model; **(D)** Decision curve of the training dataset for the predictive model; **(E)** Decision curve of the test dataset for the predictive model.

**Figure 6:**
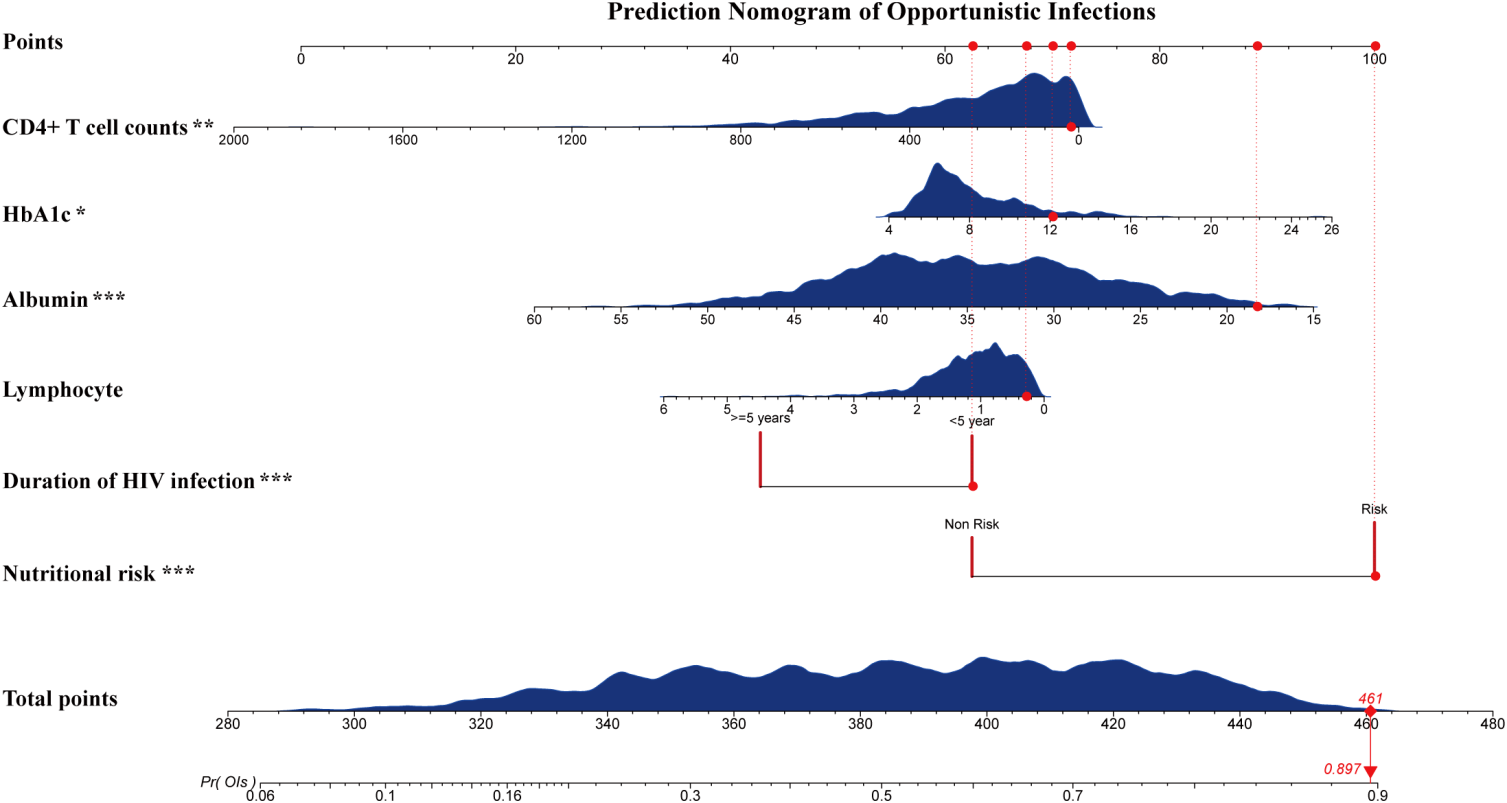
Nomograms of the predictive model for opportunistic infection. Locate the patient’s value for each variable on the corresponding axis, and draw a vertical line to the ‘Points’ axis to determine scores. Sum the scores and find the total on the ‘Total Points’ axis to assess the predicted probability of opportunistic infections. The example patient (red dots, with nutritional risk; duration of HIV infection: <5 years; Lymphocyte: 0.29(10^9/L); Albumin: 18.3 g/L; HbA1c: 12.1%; CD4+ T cell counts: 20 cells/μL) achieved a total score of 461 points, corresponding to a predicted opportunistic infections probability of 0.897. Nutritional risk, identified as ‘Non risk’ (without malnutrition risk) or ‘Risk’ (with malnutrition risk). *P<0.05, **P<0.01, ***P<0.001.

Evaluation revealed area under the curves of 0.748(95%CI:0.720-0.777) and 0.682(95%CI:0.635-0.728) for the training and test datasets, respectively (**Figure 5A**). The calibration plot showed good agreement between predicted and observed probabilities across the entire probability range in the training dataset. For the test dataset, while slight overestimation was observed in the low-probability region (0.2-0.4), the model maintained good calibration performance in both middle and high-probability regions (**Figures 5B, C**).Decision curve analysis demonstrated that the predictive model provided greater net benefits compared to the ‘treat-all’ or ‘treat-none’ strategies, with threshold probabilities ranging from 0.2 to 0.75 in the training dataset and 0.3 to 0.8 in the test dataset (**Figure 5D**).The nomogram incorporated six variables: HbA1c, CD4+T cell counts, serum albumin, lymphocyte counts, duration of HIV infection and nutritional risk. The prediction probability ranges from 0.06 to 0.90, with total points ranging from approximately 280 to 480. The example patient (red dots) achieved a total score of 461 points, corresponding to a predicted opportunistic infections probability of 0.897 (**Figure 6**).

## Discussion

With the advent of HAART and the increase in the prevalence of chronic noncommunicable diseases, the number of patients with HIV infection combined with diabetes is gradually increasing. Opportunistic infections remain a major threat to HIV-DM patients, not only increasing the medication burden (19)but also potentially delaying the initiation of antiretroviral therapy (20), which is extremely detrimental to patient prognosis. In this retrospective study, opportunistic infections in HIV-DM patients accounted for 52.01% of the total HIV-DM patients, with a mortality rate of 4.25%. The age of patients in the opportunistic infections group was greater than that in the non-opportunistic infections group, and they had a lower BMI and longer length of hospital stay consistent with previous studies (21).

In our study, serum albumin level was identified as both a significant predictor and protective factor against opportunistic infections. Previous studies have primarily focused on a low albumin level as a mortality risk factor in HIV-infected patients (22), with both low albumin level and opportunistic infections being independent predictors of poor survival (23, 24). Our findings establish a direct link between albumin level and the occurrence of opportunistic infections, suggesting a potential mechanistic pathway. Low albumin level, indicating poor nutritional status (25) and impaired immune function (26), may increase susceptibility to opportunistic infections, which could subsequently contribute to increased mortality risk. This interconnection highlights the dual clinical significance of serum albumin monitoring and maintenance in HIV-infected patients, serving as both a preventive measure against opportunistic infections and a potential determinant of survival outcomes. This finding not only demonstrates albumin’s predictive value but also emphasizes the importance of maintaining adequate nutritional status in HIV-infected patients. Additionally, our study revealed that 58.84% of hospitalized HIV-DM patients were at nutritional risk at admission (**Table 2**). Nutritional risk emerged as a crucial determinant in both the occurrence and prediction of opportunistic infections, a finding previously underexplored in the literature. These results underscore the importance of implementing routine nutritional screening during admission. And a comprehensive nutritional assessment should integrate multiple parameters, including BMI, serum albumin, total protein, and standardized nutritional assessment scales (27).

HbA1c levels are widely recognized as predictors of poor health outcomes and comorbidities, including coronary heart disease and stroke (28, 29). However, limited research has examined the relationship between HbA1c and opportunistic infections. In our study, multivariate logistic regression analyses demonstrated that HbA1c served as both a significant risk factor and a valuable predictor for opportunistic infections in HIV-DM patients. In the risk factor analysis, elevated HbA1c levels were significantly associated with increased odds of opportunistic infections (*P*<0.05). These findings were further validated in the prediction model, where HbA1c demonstrated similar predictive value. The consistency of HbA1c’s significance across both analyses strengthens the evidence for its role in opportunistic infections among HIV-DM patients. While HbA1c showed significant associations in both analyses, admission and discharge plasma glucose levels did not demonstrate such correlations. This discrepancy might be attributed to HbA1c’s ability to reflect long-term glycemic control over the preceding 2-3 months, providing a more comprehensive assessment compared to point-in-time glucose measurements (30).The relationship between elevated HbA1c and increased risk of opportunistic infections could be explained by the detrimental effects of chronic hyperglycemia on immune function and inflammatory impairment (6).Although HbA1c is widely recognized as the gold standard for long-term glycemic control assessment, its diagnostic accuracy may be compromised in the presence of anemia or hemoglobin disorders (16). Previous investigations have demonstrated a positive correlation between hemoglobin levels and HbA1c, suggesting that HbA1c-based diagnostic criteria might underestimate diabetes prevalence in HIV patients with concurrent anemia (31).A limitation of our study is that we did not specifically examine the presence of anemia among participants. Future research directions should incorporate comprehensive hematological parameters and specific diagnoses to elucidate the complex relationships between anemia, hemoglobin abnormalities, and HbA1c, as well as their collective impact on opportunistic infection occurrence. Furthermore, our findings confirmed previous observations (32–34) that acute diabetic complications serve as a prognostic risk factor (**Supplementary Table 1**). Given that these complications are strongly associated with markedly elevated HbA1c levels (>10%) (32), our results emphasize the clinical imperative for enhanced surveillance and management of patients with high HbA1c levels, both for preventing opportunistic infections and optimizing clinical outcomes.

The multivariate analyses indicated that both CD4+ T-cell counts and lymphocyte counts were significant predictors of opportunistic infections. In the prediction model, CD4+ T-cell counts showed a protective effect, which was consistently observed in the risk factor analysis. Similarly, lymphocyte counts demonstrated a protective association in the risk factor analysis. These findings align with the established understanding (35) that both CD4+ T-cell counts and lymphocyte counts serve as crucial indicators of immune function in HIV-infected patients. Duration of HIV infection was identified as a significant protective predictor against opportunistic infections, demonstrating an inverse association. For each unit increase in duration of HIV infection, the risk of opportunistic infections decreased by approximately 42.4%.This protective effect could be explained by prolonged exposure to standardized antiretroviral therapy (36) and consistent medical management (37).

The moderate association observed in our study merits careful interpretation. The odds ratio approaching 1 for CD4+ T-cell counts in the risk factor analysis, despite statistical significance, suggests limited clinical significance when considered as a continuous variable. These results suggest that small changes in CD4+ T-cell counts may have limited immediate impact. But sustained abnormal levels might significantly affect clinical outcomes. During model development, we attempted to incorporate CD4T cell counts as a categorical variable. However, this categorization diminished the statistical significance of other clinically relevant predictors, notably HbA1c. This finding suggests potential interactions between variables and underscores the importance of maintaining the continuous nature of certain predictors to preserve their predictive value. Additionally, the inconsistent significance of lymphocyte counts between the risk factor analysis (P<0.05) and prediction model (P>0.05) highlights the complexity of using immunological parameters as predictive markers (38).While lymphocyte counts has been investigated as a potential surrogate marker in certain clinical settings (39), its predictive value may vary depending on the specific context and outcome measures being assessed.

In contrast to previous studies (15), our findings revealed that despite a higher prevalence of comorbidities in the opportunistic infection group compared to the non-opportunistic infection group, comorbidities were not identified as a risk factor for opportunistic infections among HIV-DM patients.

Our findings have important implications for clinical practice guidelines and regional healthcare policies regarding opportunistic infection management in HIV-DM patients. First, the prediction model could be integrated into existing clinical guidelines (40) to provide standardized risk assessment protocols for this specific patient population. This would enable healthcare providers to implement more targeted preventive strategies based on individual risk profiles. Second, our risk prediction approach could inform the development of regional screening protocols, helping to optimize resource allocation in different healthcare settings (41). Patients identified as high-risk by the model might benefit from more frequent monitoring and enhanced preventive measures, while those at lower risk could follow standard care protocols. Furthermore, the identified risk factors and their relative importance could guide the development of preventive healthcare policies, particularly in regions with high prevalence of HIV-DM comorbidity. Healthcare systems could use this information to establish more effective surveillance programs and allocate resources more efficiently for opportunistic infection prevention (42).

## Limitation

There are some limitations of this study. First, while multiple imputation was utilized to handle missing data, we acknowledged that potential bias might persist, which could influence the internal validity of our findings despite our best methodological efforts. Second, the predictive performance of our model was moderate, suggesting room for improvement in discriminative ability. Future studies could consider incorporating additional clinical biomarkers and baseline characteristics to enhance the model’s predictive performance. Potential improvements include integrating comprehensive immunological parameters (e.g., CD19, CD56 cell counts, and inflammatory markers), incorporating sensitive diabetes-related indicators (e.g., glycated albumin), and accounting for medication adherence and treatment history. Furthermore, the adoption of more advanced machine learning techniques and larger sample sizes might help improve both model discrimination and calibration. Finally, this study was conducted as a single-center retrospective analysis, which inherently carries methodological limitations such as potential selection bias and incomplete data capture. As a retrospective analysis, causative relationships cannot be firmly established, and there may be residual confounding factors that we have not accounted for, such as unmeasured lifestyle variables or socioeconomic factors. To validate and generalize our findings, future research should employ multi-center prospective cohort designs with larger sample sizes, which would provide more robust evidence and potentially better account for regional variations in patient characteristics and treatment practices.

## Conclusion

This study identified significant risk factors for opportunistic infections through multifactorial logistic regression analysis, including nutritional risk, duration of HIV infection, HbA1c levels, lymphocyte counts, albumin level, and CD4+ T cell counts. Based on these clinical indicators, we developed a predictive model for HIV-DM hospitalized patients. Our findings provide a scientific framework for the early identification and prevention of opportunistic infections in this vulnerable population, with the ultimate goal of improving patient outcomes and quality of life.

## Data Availability

The raw data supporting the conclusions of this article will be made available by the authors, without undue reservation.
