# Supplementary figures and images for: Nutritional risk and HbA1c as critical risk factors and predictors of opportunistic infections in HIV-DM comorbid patients: a retrospective cross-sectional study
Source: Front Endocrinol (Lausanne). 2025 Jan 10;15:1527936. doi: 10.3389/fendo.2024.1527936 (PMC11757115; doi:10.3389/fendo.2024.1527936)

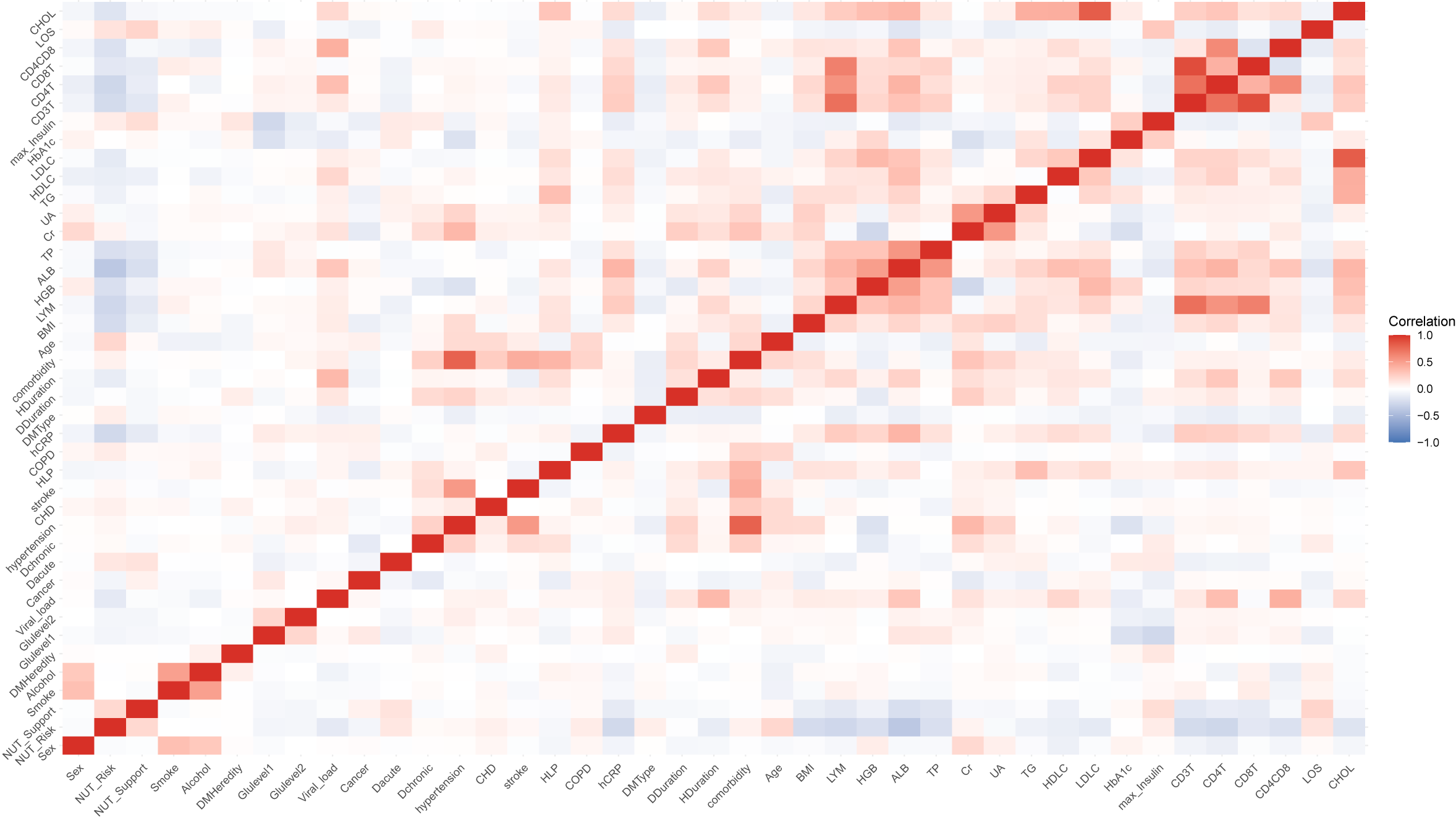

Supplement: Supplementary file 1 [file Image1.tif]

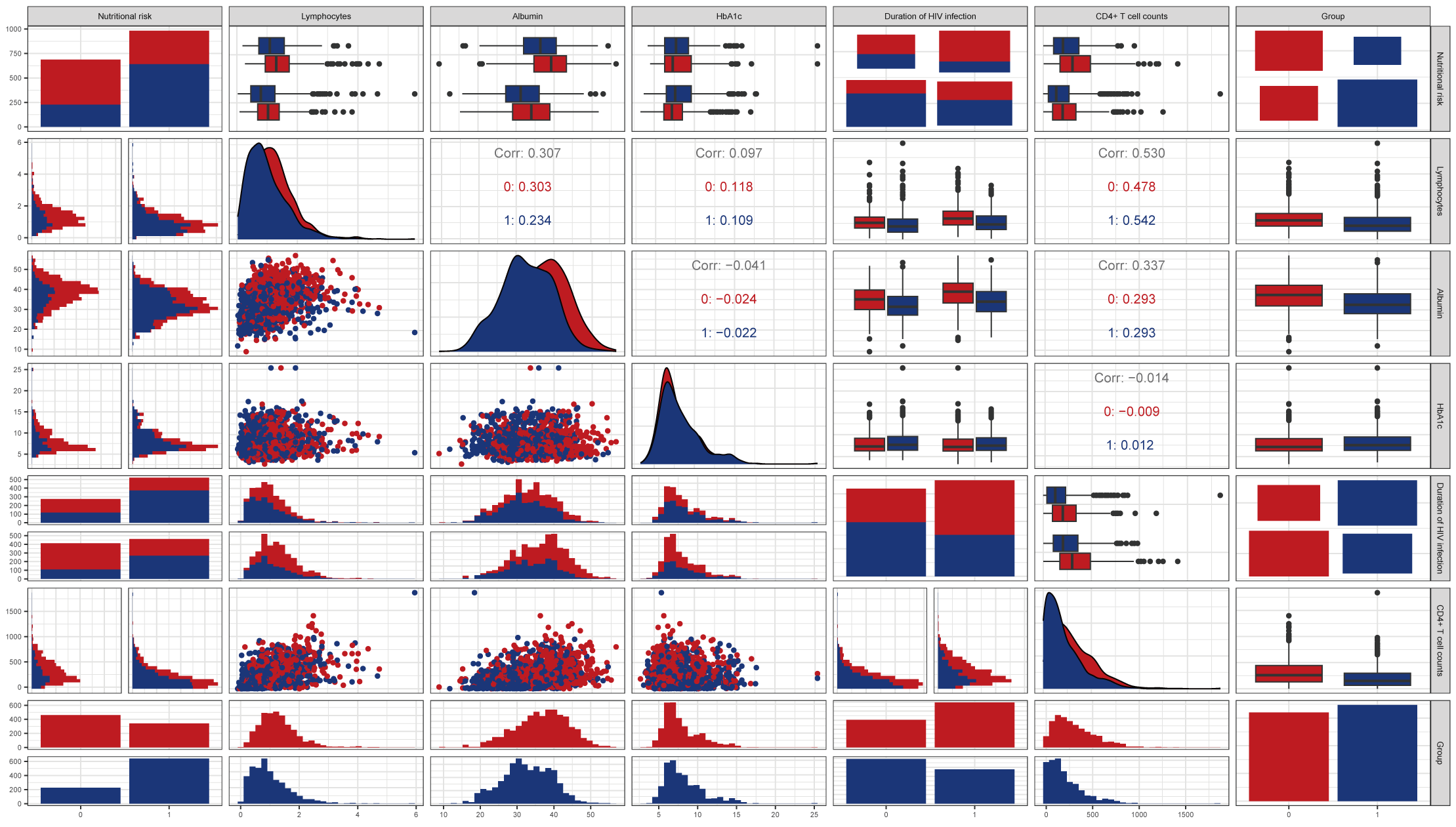

Supplement: Supplementary file 2 [file Image2.tif]
