# Supplementary material for: Nutritional risk and HbA1c as critical risk factors and predictors of opportunistic infections in HIV-DM comorbid patients: a retrospective cross-sectional study
Source: Front Endocrinol (Lausanne). 2025 Jan 10;15:1527936. doi: 10.3389/fendo.2024.1527936 (PMC11757115; doi:10.3389/fendo.2024.1527936)
Supplement: Supplementary file 3 [file Table1.docx]

Supplementary Table 1 Results of multivariate logistic regression of risk factor analysis model for prognosis

| **Variable** | **B** | **Wald** | **Std. Error** | **OR (95%CI)** | ***P*** |
| --- | --- | --- | --- | --- | --- |
| Nutritional risk | -3.208 | 9.99 | 1.015 | 0.04(0.002,0.187) | 0.002 |
| Nutritional Support | -0.892 | 11.416 | 0.264 | 0.41(0.244,0.688) | < 0.001 |
| Acute diabetes complications | -0.96 | 8.291 | 0.333 | 0.383(0.203,0.753) | 0.004 |
| Duration of HIV infection | 0.709 | 5.83 | 0.294 | 2.032(1.16,3.688) | 0.016 |
| Uric acid | -0.002 | 11.222 | 0.001 | 0.998(0.997,0.999) | < 0.001 |
| CD4 T cell counts | 0.004 | 11.809 | 0.001 | 1.004(1.002,1.006) | < 0.001 |


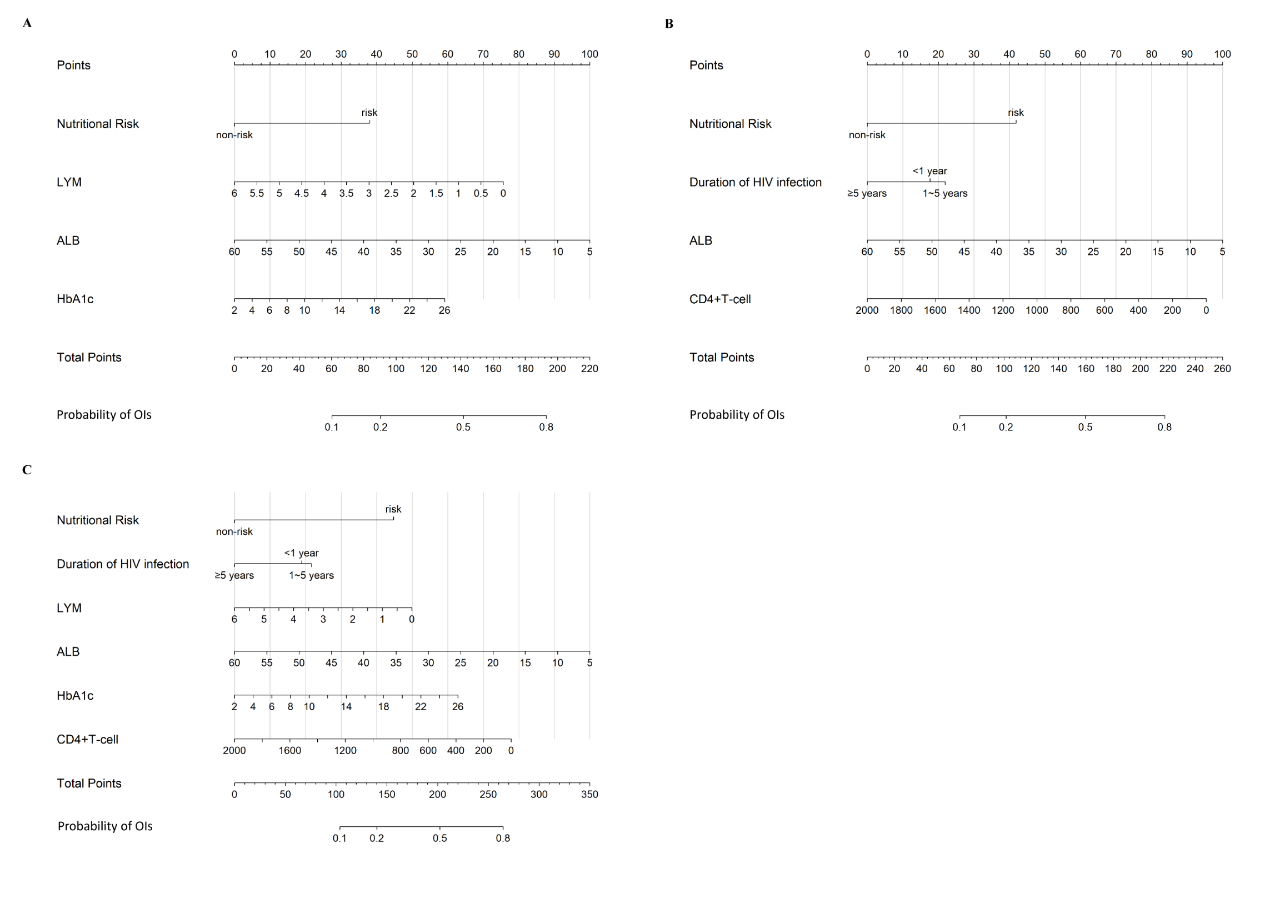


This figure was deleted.

**~~Supplementary figure 1.~~** ~~Nomograms of opportunistic infections. (A)Model 1, independent variables identified by multivariate logistic regression following initial univariate logistic regression screening;(B)Model 2, independent variables selected initially via LASSO regression and subsequently finalized based on multivariate logistic regression analysis;(C)Model 3 was composed of the independent variables of model 1 and model 2.~~

**SUPPLEMENTARY FIGURE LEGENDS**

**Supplementary Figure 1.** Correlation heatmap of all variables. NUT_Risk: Nutritional_risk; DMHeredity: Family history of diabetes; Glulevel1: Blood Glucose Target Achievement at Admission; Glulevel2:Blood Glucose Target Achievement at Discharge; Dacute: Acute Diabetes Complications; Dchronic: Chronic Diabetes Complications; CHD: Coronary heart disease; HLP: Hyperlipidaemia; COPD: Chronic obstructive pulmonary disease; DMType: Diabetes Type; DDuration: Duration of diabetes; HDuration: Duration of HIV infection; LYM: Lymphocytes; HGB: Hemoglobin; ALB: Albumin ; TP: Total protein; Cr: Creatinine; UA: Uric acid; TG: Triglyceride; HDLC: High-Density Lipoprotein Cholesterol; LDLC: Low-Density Lipoprotein Cholesterol; max_Insulin: Maximum dose of Insulin; CD3T: CD3+T-cell counts; CD4T: CD4+T-cell counts;CD8T: CD8+T-cell counts;CD4CD8: CD4/CD8 T-Cell Ratio; LOS: Length of hospital stay; CHOL: Cholesterol.

**Supplementary Figure 2.** Pairs plot of key clinical indicators with distributions and correlations. Corr: Correlation coefficient.
